# Clinical characterization and healthcare burden of difficult-to-treat inflammatory bowel disease in Latin America: a real-world registry-based study

**DOI:** 10.1093/crocol/otag058

**Published:** 2026-06-13

**Authors:** Jesus K Yamamoto-Furusho, Renata Fróes, Cristina Flores, Domingo Balderramo, Juan Sebastian Lasa, Tárcia Nogueira Ferreira Gomes, Sócrates Bautista Martínez, Guillermo Veitia, Beatriz Iade-Vergara, Norma Nathaly Parra-Holguín, Amanda Yaeza Ferreira, Milene Fernandes, Vera Vicente, Fabian Juliao-Baños

**Affiliations:** Inflammatory Bowel Disease Clinic, Department of Gastroenterology, Instituto Nacional de Ciencias Médicas y Nutrición Salvador Zubirán, Mexico City, Mexico; Gastroenterology Department, GastromedRJ Clinic, Rio de Janeiro, Brazil; Inflammatory Bowel Disease and Immunomediated Disease Center—DIIMUNO, Porto Alegre, Brazil; Gastroenterology and Endoscopy Department, Hospital Privado Universitario de Córdoba, Córdoba, Argentina; Gastroenterology Department, Center for Medical Education, Clinical Research Norberto Quirno (CEMIC), Buenos Aires, Argentina; Immunology LATAM, Johnson & Johnson, São Paulo, Brazil; Gastroenterology Center, Centros de Diagnóstico y Medicina Avanzada y de Conferencias Médicas y Telemedicina (CEDIMAT), Santo Domingo, Dominican Republic; Gastroenterology Department, Hospital Vargas de Caracas, Caracas, Venezuela; Gastroenterology Department, Centro de Asistencia del Sindicato Médico del Uruguay (CASMU), Montevideo, Uruguay; Inflammatory Bowel Disease Clinic, Department of Gastroenterology, Instituto Nacional de Ciencias Médicas y Nutrición Salvador Zubirán, Mexico City, Mexico; Immunology LATAM, Johnson & Johnson, São Paulo, Brazil; CTI—Clinical Trial & Consulting Services, Lisboa, Portugal; CTI—Clinical Trial & Consulting Services, Lisboa, Portugal; Inflammatory Bowel Disease Clinic, Hospital Pablo Tobón Uribe, Medellín, Colombia

**Keywords:** difficult-to-treat, inflammatory bowel disease, Latin America, epidemiology

## Abstract

**Background:**

Inflammatory bowel disease (IBD) is increasing across Latin America (LATAM). We aimed to determine the frequency and characteristics of difficult-to-treat IBD (DTT-IBD) in this region, using international consensus criteria.

**Methods:**

A retrospective study using data from IBD registries across 11 LATAM countries: the EPILATAM-IBD study (9 countries), the Brazilian national IBD registry, and 2 Argentinian single-center registries. Adults with ulcerative colitis (UC), Crohn’s disease (CD), or unclassified IBD were included. DTT-IBD was defined as meeting at least one of the following criteria: failure of ≥2 advanced mechanisms of action, chronic antibiotic-refractory pouchitis, CD recurrence after ≥2 resections, or complex perianal CD. Comparisons between DTT and non-DTT groups were performed using Mann-Whitney and Chi-square/Fisher exact tests (*P* < .05).

**Results:**

Among 5699 patients with complete data [40% CD, median disease duration 9 years (Q1–Q3: 5-15)], 747 (13%) met DTT-IBD criteria. DTT prevalence was higher with CD (30%) than among patients with UC (2%). Complex perianal disease predominated in DTT-CD (91%), whereas failure of ≥2 advanced mechanisms of action predominated in DTT-UC (93%). Compared to non-DTT patients, those with DTT-IBD were younger at symptom onset [median age 24 (19–34) years] and IBD diagnosis [26 (21–37) years], and had longer time to diagnosis (*P*-value<.001). DTT-CD was associated with penetrating behavior (39%) and ileocolonic disease (46%); DTT-UC with extensive colitis (83%).

**Conclusions:**

DTT-IBD affected 13% of patients treated in LATAM, with nearly one-third of individuals with CD presenting more severe phenotypic features. These findings highlight the need for comprehensive IBD care strategies in this complex patient population.

## Graphical Abstract

**Figure:**
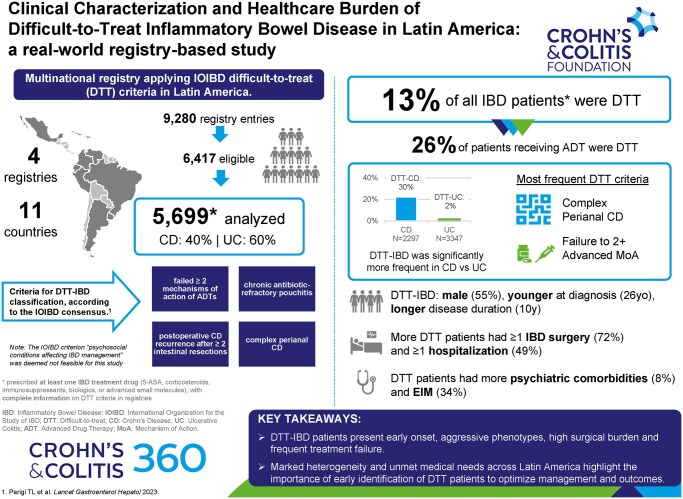

## Introduction

Inflammatory bowel disease (IBD) is a chronic, relapsing disorder characterized by inflammation of the gastrointestinal tract. There are two main subtypes of IBD, ulcerative colitis (UC) and Crohn’s disease (CD), associated with significant clinical burden and impact on quality of life.^1^^,^^2^ In recent decades, the incidence and prevalence of IBD have been increasing in Latin American countries, a region where access to healthcare and advanced therapies remains heterogeneous.^3^^,^^4^ Despite advances in therapy, current treatments for IBD still have limitations, and there are no curative or preventive options available. In the context of longstanding disease, physicians estimate that only 37% to 55% of their patients with moderate to severe CD or UC achieve remission with biologic therapy.^5^ It has been suggested that a substantial proportion of IBD patients may present with difficult-to-treat (DTT) disease.^6^^,^^7^ The International Organization for the Study of IBD (IOIBD) recently defined DTT-IBD as the failure of at least two mechanisms of action of biologics, or advanced small molecules, CD postoperative recurrence after 2 or more intestinal resections, chronic antibiotic-refractory pouchitis, complex perianal CD, or coexisting psychosocial issues impairing adequate clinical management.^8^ These new criteria aim to provide a simple and operational definition of DTT-IBD to standardize future research and guide treatment choice.^8^ However, the epidemiological and clinical features and impact of DTT-IBD on healthcare utilization are to be characterized, and, to our knowledge, no large real-world study has described DTT-IBD in Latin America.^4^

Within this context, our objective was to evaluate the epidemiological and clinical characteristics of DTT-IBD and elucidate the burden of DTT-IBD in Latin American countries, based on existing IBD registries. This study may guide future research and treatment strategies for this challenging condition.

## Methods

### Study design, data sources, and participants

This was a noninterventional, retrospective, cross-sectional study based on secondary data from 4 different LATAM registries, involving 11 countries (Argentina, Brazil, Colombia, Cuba, Dominican Republic, Ecuador, Mexico, Peru, Puerto Rico, Uruguay, and Venezuela).^9^^,^^10^ Eligible patients for this analysis were adults (≥18 years old) at the time of registry inclusion, with a confirmed diagnosis of IBD (UC, CD, or unclassified IBD) based on the medical assessment that considers clinical, biochemical, endoscopic, histopathological, and radiological features, and having at least one drug prescribed for IBD treatment (5-aminosalicylates, corticosteroids, immunosuppressants, biologics, or advanced small molecules—JAK inhibitor or S1P modulator). There were no exclusion criteria. In addition, patients had been enrolled consecutively in the source registries during routine outpatient appointments with gastroenterologists or proctologists, irrespective of follow-up time at the center.

### Case definition and study variables

Patients were classified as having DTT‑IBD if they met at least one IOIBD criterion, operationalized through standardized proxies (ie, surrogate variables) adapted to the structure and data availability of each registry (Table  1). This approach was implemented after recognizing that our real-world large registry data did not directly capture the IOIBD criteria, as in similar studies of other conditions.^12^

**Table 1:** Definition of DTT-IBD criteria and corresponding proxies by source.

**Criteria of DTT-IBD** ^8^	Proxy in EPILATAM-IBD	Proxy in GEDIIB	Proxy in CEMIC	Proxy in HPUC
**Failure of biologics and/or advanced small molecules with at least 2 different mechanisms of action defines DTT-IBD**	3+ ADT of at least 2 mechanisms of action	3+ ADT of at least 2 mechanisms of action OR Failure to at least 2 ADTs of 2 different mechanisms of action^a^	3+ ADT of at least 2 mechanisms of action	3+ ADT of at least 2 mechanisms of action
**Postoperative recurrence of CD after 2 or more intestinal resections defines DTT-CD**	2+ intestinal resections + postoperative recurrence CD OR 3+ intestinal resections	3+ intestinal resections	2+ intestinal resections + postoperative recurrence CD OR 3+ intestinal resections	2+ intestinal resections + postoperative recurrence CD OR 3+ intestinal resections
**Chronic antibiotic-refractory pouchitis defines DTT-IBD**	Pouchitis with surgical treatment	Pouchitis with surgical treatment	Pouchitis with surgical treatment	Pouchitis with surgical treatment
**Complex perianal Crohn’s disease defines DTT-CD^b^**	Perianal disease OR CD + any surgical procedure regarding perianal fistulas or abscess	Modifier “p” OR CD + any surgical procedure regarding perianal abscess or fistulas OR Complex perianal fistula^c^	Modifier “p” OR CD + any surgical procedure regarding perianal abscess or fistulas	Modifier “p” OR CD + any surgical procedure regarding perianal abscess or fistulas OR Complex perianal fistula**

Abbreviations: ADT, advanced drug therapy; CD, Crohn’s disease; CEMIC, Centro de Educación Médica y Investigaciones Clínicas “Norberto Quirno”; DTT, difficult-to-treat; EPILATAM-IBD, Epidemiologic Characterization of Inflammatory Bowel Disease in Latin America Study; GEDIIB, Brazilian Organization of Crohn’s Disease and Colitis; HPUC, Hospital Privado Universitário de Córdoba; IBD, inflammatory bowel disease; UC, ulcerative colitis.

a The variable “discontinued ADT due to failure” was collected in GEDIIB (423 ADTs discontinued among 304 patients with ADT discontinuation).

b Complex perianal fistulas: a fistula meeting any of the following criteria: high location (high inter sphincteric, high trans sphincteric, extra sphincteric, or supra sphincteric), multiple external openings, perianal abscess, anal stenosis, or proctitis.^11^

c The variable “complex perianal fistula” was collected in GEDIIB (*n* = 115 patients) and HPUC (*n* = 16 patients).

Our study proxies were developed using all relevant information from each database and validated by clinical experts from the LATAM region:

- The IOIBD criterion “failure of biologics, or advanced small molecules with ≥2 mechanisms of action” translated to the proxy “patients with ≥3 advanced therapies (ADT) of at least 2 mechanisms of action,” reflecting the likelihood that multiple switches indicated treatment failure or loss of response. In registries where “reason for stopping drug” was available, patients with documented failure of 2 ADTs from different mechanisms of action were also identified.
- The criterion “postoperative recurrence of CD after 2 or more intestinal resections” was operationalized as the proxy “patients with 3 or more intestinal resections,” based on the clinical assumption that repeated resections typically reflect recurrent postoperative disease.
- For the criterion “chronic antibiotic‑refractory pouchitis,” the proxy was “patients who had undergone surgical treatment for pouchitis,” given the absence of standardized data on antibiotic response across registries.
- The proxy for the IOIBD criterion “Complex perianal CD” was defined using the Montreal “p” modifier, recorded perianal disease phenotype, or documented perianal fistula/abscess surgery. According to the clinical perspective, the modifier “p” would be added mostly in complex cases of perianal disease. When available, registry‑captured “complex perianal fistula” was also incorporated.^11^
- Finally, the criterion “patient coexisting psychosocial issues that impair adequate clinical management” was deemed too subjective to be operationalized in this study, as described for other diseases (eg, rheumatoid arthritis).^12^

All registries contributed de-identified data, harmonized into a common model. The collected variables included sociodemographics; smoking status; family history; comorbidities; age at IBD diagnosis and symptom onset; CD location and phenotype at diagnosis; UC extent at diagnosis; extraintestinal manifestations (EIMs); relapses and complications since diagnosis; IBD-related surgeries and hospitalizations; pharmacologic treatments with indicators of steroid dependence/resistance, and refractory disease.

### Statistical analysis

This was an exploratory, descriptive study; therefore, no a priori statistical hypotheses were defined. All patients meeting eligibility criteria were included, without imputation of missing data. The proportion of DTT-IBD patients was reported as percentage with 95% confidence interval (CI). Group comparisons were conducted using the Mann–Whitney test for continuous variables and the Chi‑square or Fisher’s exact test for categorical variables, depending on test assumptions. To identify factors independently associated with DTT‑CD and DTT‑UC, we performed multivariable logistic regression with backward stepwise selection. Candidate variables included: sex, referral institution, age at IBD diagnosis, age at symptom onset, and time between symptom onset and diagnosis. For the DTT-CD model, CD location and phenotype at diagnosis were included; for the DTT-UC model, UC extent at diagnosis was included. All statistical tests were two-sided with a 5% significance level. Analyses were performed by CTI Clinical Trial and Consulting Services, using SAS^®^ software, version 9.4 (SAS Institute Inc, Cary, USA).

### Ethical considerations

This study used anonymized secondary data from existing IBD registries in Latin America. All participating registries obtained prior approval from their respective institutional review boards or ethics committees, in accordance with the Declaration of Helsinki and local regulations. As this was a noninterventional, retrospective study using de-identified data, informed consent was waived or not required by the ethics committees. No patient-identifiable information was accessed or disclosed at any stage of the study.

## Results

### Sample characteristics

Data from the Latin American registries included 9280 patients, of whom 70.1% (*n* = 6417) were deemed eligible for the analysis (Table S1). Overall, 3572 (55.7%) eligible patients had UC, and 2778 (43.4%) had CD. Most were from Brazil (57.0%) and Mexico (19.6%).

Among eligible patients, 5699 (88.8%) had complete information on DTT criteria (Table 2). This subgroup was predominantly female (55.9%), referred from private institutions (63.0%), nonsmokers (88.2%), and without a family history of IBD (98.0%). The median disease duration was 9 years, with similar ages at diagnosis and symptom onset. Among CD patients with complete DTT information (*n* = 2297), the ileocolonic location was most frequent (43.2%), followed by ileal and colonic involvement, while isolated upper gastrointestinal disease was uncommon. At diagnosis, the inflammatory phenotype was most frequent (52.6%), with smaller proportions presenting with stricturing (27.5%) or penetrating disease (19.9%). For UC patients with complete DTT information (*n* = 3347), extensive colitis was the most common extent at diagnosis (49.1%), followed by left‑sided colitis and proctitis.

**Table 2:** Demographic and clinical characteristics of DTT and non-DTT patients.

	Overall (*N* = 5699)	*N*	DTT group (*N* = 747)	*n*	Non-DTT group (*N* = 4952)	*n*	p-value^a^
**Type of IBD, *n* (%)**							
** UC**	3347 (58.7)	5699	54 (7.2)	747	3293 (66.5)	4952	
** CD**	2297 (40.3)		693 (92.8)		1604 (32.4)		
** Unclassified IBD**	55 (1.0)		0 (0.0)		55 (1.1)		
**Age at IBD diagnosis (years), median (Q1-Q3)**	33 (24-46)	5500	26 (21-37)	719	35 (25-48)	4780	**<.001**
**Disease duration (years), median (Q1-Q3)**	9 (5-15)	5503	10 (6-16)	722	9 (5-15)	4781	**<.001**
**Disease duration, *n* (%)**							
** <5 years**	1200 (21.8)		128 (17.7)		1072 (22.4)		**.006**
** 5-10 years**	2027 (36.8)		264 (36.6)		1763 (36.9)		
** >10 years**	2276 (41.4)		330 (45.7)		1946 (40.7)		
**Age at symptom onset (years), median (Q1-Q3)**	32 (23-45)	5327	24 (19-34)	688	33 (24-46)	4639	**<.001**
**Time between onset of symptoms and diagnosis (years), mean, median (Q1-Q3)**	1.22, 0 (0-1)	5297	1.70, 0 (0-2)	682	1.15, 0 (0-1)	4615	**<.001**
**Female sex, *n* (%)**	3183 (55.9)	5696	338 (45.2)	747	2845 (57.5)	4949	**<.001**
**Family history (1st grade) of IBD, *n* (%)**	57 (2.0)	2816	11 (5.4)	203	46 (1.8)	2613	**.002^b^**
**Current smokers, *n* (%)**	639 (11.8)	5436	52 (7.4)	699	587 (12.4)	4737	**<.001**
**Private referral institution, *n* (%)**	2385 (63.0)	3786	305 (73.3)	416	2080 (61.7)	3370	**<.001**
**CD location at diagnosis, *n* (%)**		2184		649		1535	**<.001**
** Ileal^c^**	697 (31.9)		148 (22.8)		549 (35.8)		
** Colonic^c^**	497 (22.8)		189 (29.1)		308 (20.1)		
** Ileocolonic^c^**	944 (43.2)		298 (45.9)		646 (42.1)		
** Upper gastrointestinal tract isolated**	46 (2.1)		14 (2.2)		32 (2.1)		
**CD phenotype at diagnosis, *n* (%)**		2050		611		1439	**<.001**
** Nonstricturing, nonpenetrating (B1)**	1079 (52.6)		247 (40.4)		832 (57.8)		
** Stricturing (B2)**	564 (27.5)		127 (20.8)		437 (30.4)		
** Penetrating (B3)**	407 (19.9)		237 (38.8)		170 (11.8)		
**UC extension at diagnosis, *n* (%)**		3280		52		3228	**<.001**
** Extensive colitis (E3)**	1611 (49.1)		43 (82.7)		1568 (48.6)		
** Left-sided colitis (E2)**	904 (27.6)		8 (15.4)		896 (27.8)		
** Proctitis (E1)**	765 (23.3)		1 (1.9)		764 (23.7)		

Percentages are based on patients with information (*n* column). Overall patients include UC, CD, and unclassified IBD patients. *P*-values presented in bold indicate statistical significance.

Abbreviations: CD, Crohn’s disease; DTT, difficult-to-treat; IBD, inflammatory bowel disease; *n*, number of patients without missing information; Q1, first quartile; Q3, third quartile; UC, ulcerative colitis.

a *P-*value from the Mann-Whitney test, for quantitative variables, and chi-square test for qualitative variables, except

b Fisher exact test.

c With or without involvement of upper gastrointestinal tract—CD location by overall patients: ileal + upper gastrointestinal *n* = 21, colonic + upper gastrointestinal tract *n* = 5, ileocolonic + upper gastrointestinal tract *n* = 17.

### DTT-IBD frequency and criteria

**Figure 1:**
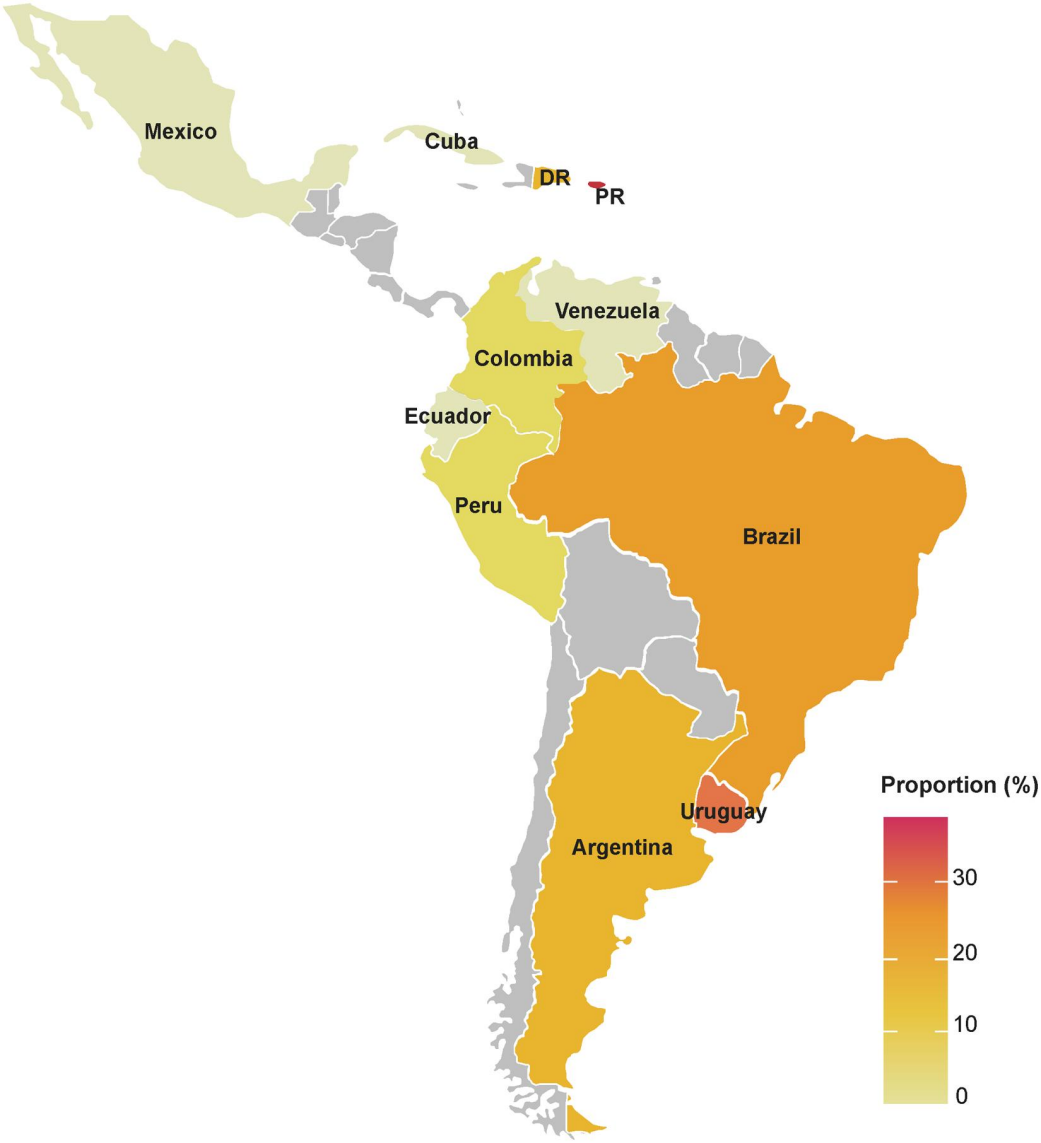
Proportion of DTT-IBD patients by country.

Overall, 747 patients (13.1%, 95% CI [12.2%; 14.0%]) met criteria for DTT‑IBD, with variation across countries (Figure  1). The highest proportions were observed in Puerto Rico (38.6%), Uruguay (27.8%), and Brazil (18.7%). DTT‑IBD was significantly more common in CD (30.2%) than among UC patients (1.6%). Patients with DTT‑CD (*n* = 693) met most frequently the “complex perianal disease” criterion (90.5%), followed by “failure of 2 or more ADT mechanisms” (8.7%), “postoperative recurrence” (5.5%), and “refractory pouchitis” (1.2%) (Table S1). Excluding the “complex perianal disease” criterion, 104 (5.3%) CD patients met the remaining DTT criteria. Among patients with DTT‑UC (*n* = 54), nearly all met the criterion “failure of 2 or more ADT mechanisms” (92.6%), with “refractory pouchitis” identified in 4 patients (7.4%).

### Factors associated with DTT-IBD

Compared to non-DTT patients, statistically significantly more DTT patients were male (54.8%), referred from private institutions (73.3%), with a family history of IBD (5.4%), and with no current smoking habits (92.6%) (Table 2). DTT patients were younger at symptom onset and diagnosis, had longer disease duration (more than 10 years: 45.7%), and had longer time until IBD diagnosis.

Among CD patients, those with DTT-CD presented statistically significantly more colonic (29.1%) and ileocolonic (42.1%) locations and a penetrating CD phenotype (38.8%). After adjusting for the main sociodemographic and CD clinical characteristics at diagnosis, patients with colonic location had higher odds (vs. ileocolonic patients) of being identified with DTT-CD (OR = 1.96, 95% CI [1.42; 2.72]), while older age at symptom onset (OR = 0.97, 95% CI [0.96; 0.98]) and female sex (OR = 0.71, 95% CI [0.55; 0.93]) were associated with lower odds of DTT-CD (Table S2).

Extensive colitis was statistically significantly more frequent among DTT-UC patients (82.7%) than non-DTT UC patients. Being referred from a private institution remained significantly associated with higher odds of DTT-UC (OR = 3.65, 95% CI [1.50; 8.84]), after adjusting for the main sociodemographic and UC characteristics at diagnosis (Table S3).

### Comorbidities, complications, and EIMs associated with DTT-IBD

Compared with non-DTT patients, DTT patients showed a distinct comorbidity profile (Table 3). They had significantly fewer musculoskeletal comorbidities (4.7%), but higher rates of psychiatric (7.7%), hepatic/gastrointestinal (7.4%), and urogenital conditions (4.4%). Among DTT patients, the most frequent IBD complications were perianal fistula/abscess (52.0%), fatigue (13.7%), anemia (8.2%), infection (7.0%), and abdominal fistula/abscess (5.7%). All of these complications—except anemia, which was more common in non‑DTT patients—occurred significantly more often in the DTT group.

**Table 3:** Comorbidities, complications, and EIMs of DTT and non-DTT patients.

	Overall (*N* = 5699)	DTT group (*N* = 747)	Non-DTT group (*N* = 4952)	*P*-value^a^
**Any comorbidity, *n* (%)**	*n* = 5133	*n* = 690	*n* = 4443	.859
** No**	2999 (58.4)	401 (58.1)	2598 (58.5)	
** Yes**	2134 (41.6)	289 (41.9)	1845 (41.5)	
**Comorbidities, *n* (%)^c^**				
** Musculoskeletal**	401 (7.2)	35 (4.7)	366 (7.5)	**.005**
** Cardiovascular**	842 (16.1)	96 (13.7)	746 (16.5)	.064
** Neurologic**	73 (1.5)	8 (1.2)	65 (1.5)	.514
** Psychiatric**	191 (4.0)	49 (7.7)	142 (3.5)	**<.001**
** Skin**	128 (2.6)	24 (3.6)	104 (2.5)	.082
** Hepato/gastrointestinal**	250 (5.3)	47 (7.4)	203 (5.0)	**.011**
** Hematologic**	48 (1.0)	9 (1.4)	39 (0.9)	.290
** Urogenital**	106 (2.2)	28 (4.4)	78 (1.9)	**<.001**
** Respiratory**	86 (1.8)	10 (1.6)	76 (1.9)	.621
** Endocrine**	702 (13.5)	96 (13.7)	606 (13.4)	.814
** Oncology**	99 (1.9)	17 (2.5)	82 (1.9)	.249
** Other**	293 (5.8)	43 (6.4)	250 (5.7)	.467
**Any IBD complication since diagnosis, *n* (%)**	*n* = 5457	*n* = 717	*n* = 4740	<.001
** No**	3745 (68.6)	166 (23.2)	3579 (75.5)	
** Yes**	1712 (31.4)	551 (76.8)	1161 (24.5)	
**Complications of IBD, *n* (%)^c^**				
** Abdominal fistula/abscess**	95 (1.8)	41 (5.7)	54 (1.1)	**<.001**
** Perianal fistula/abscess**	395 (7.3)	372 (52.0)	23 (0.5)	**<.001**
** Toxic megacolon**	16 (0.3)	1 (0.1)	15 (0.3)	.711^b^
** Intestinal obstruction/perforation**	120 (2.2)	41 (5.7)	79 (1.7)	**<.001**
** Colorectal cancer**	23 (0.4)	3 (0.4)	20 (0.4)	1.000^b^
** Infection**	142 (2.6)	50 (7.0)	92 (2.0)	**<.001**
** Thromboembolic events**	91 (1.6)	13 (1.8)	78 (1.6)	.721
** Anemia**	607 (11.4)	58 (8.2)	549 (11.8)	**.005**
** Fatigue**	522 (9.2)	102 (13.7)	420 (8.5)	**<.001**
** Other**	123 (2.2)	34 (4.7)	89 (1.9)	<.001
**Any extraintestinal manifestation, *n* (%)**	*n* = 5699	*n* = 747	*n* = 4952	<.001
** No**	4108 (72.1)	494 (66.1)	3614 (73.0)	
** Yes**	1591 (27.9)	253 (33.9)	1338 (27.0)	
**Extraintestinal manifestations, *n* (%)^c^**				
** Articular**	1337 (23.5)	207 (27.7)	1130 (22.8)	**.003**
** Arthralgia/arthritis**	1130 (19.8)	183 (24.5)	947 (19.1)	**.001**
** Axial articular**	364 (6.4)	49 (6.6)	315 (6.4)	.836
** Sacroiliitis**	106 (1.9)	13 (1.7)	93 (1.9)	.762
** Ankylosing spondylitis**	279 (5.0)	36 (4.8)	243 (5.0)	.860
** Primary sclerosing cholangitis**	116 (2.0)	10 (1.3)	106 (2.1)	.148
** Skin**	149 (2.6)	30 (4.0)	119 (2.4)	**.010**
** Pyoderma gangrenosum**	56 (1.0)	8 (1.1)	48 (1.0)	.819
** Erythema nodosum**	91 (1.6)	22 (3.0)	69 (1.4)	**.002**
** Uveitis**	123 (2.2)	25 (3.3)	98 (2.0)	**.016**
** Oral ulcers**	114 (2.0)	34 (4.6)	80 (1.6)	**<.001**

Percentages are based on patients with information (*n* column). Overall patients include UC, CD, and unclassified IBD patients. *P*-values presented in bold indicate statistical significance.

Abbreviations: DTT, difficult-to-treat; IBD, inflammatory bowel disease.

a *P-*value from the chi-square test except

b Fisher exact test.

c More than one possible option per patient.

DTT patients also had higher frequencies of EIMs, including arthralgia/arthritis (24.5%), erythema nodosum (3.0%), uveitis (3.3%), and oral ulcers (4.6%), compared with non‑DTT patients.

### IBD-related surgeries and hospitalizations

DTT patients underwent significantly more IBD‑related surgeries than non‑DTT patients (Table 4). A majority (72.4%) of DTT patients had at least 1 surgery, of whom 22.4% had 3 or more surgeries. DTT patients also experienced significantly higher rates of both perianal (52.2%) and abdominal (31.0%) surgeries, including ostomies and abdominal drainages.

**Table 4:** IBD surgeries and hospitalizations: DTT and non-DTT patients.

	Overall (*N* = 5699)			DTT group (*N* = 747)			Non-DTT group (*N* = 4952)			*P*-value^a^
**Any surgery since diagnosis, *n* (%)**	1034 (18.2)		*n* = 5692	536 (72.4)		*n* = 740	498 (10.1)		*n* = 4952	**<.001**
**Number of surgeries**			*n* = 969			*n* = 487			*n* = 482	**<.001**
** Mean ± standard deviation**	1.57 ± 1.04			1.84 ± 1.29			1.31 ± 0.61			
** Median [min-max]**	1.00 [1-9]			1.00 [1-9]			1.00 [1-4]			
** 1**	648 (66.9%)			281 (57.7%)			367 (76.1%)			—
** 2**	184 (19.0%)			97 (19.9%)			87 (18.0%)			
** 3+**	137 (14.1%)			109 (22.4%)			28 (5.9%)			
**Type of surgery^b^, *n* (%)**										
** Abdominal**	691 (12.3)			214 (31.0)			477 (9.6)			**<.001**
** Resection (small bowel and/or colon)**	636 (92.0)			196 (91.6)			440 (92.2)			.769
** Colectomy**	488 (70.6)			159 (74.3)			329 (69.0)			.155
** Ostomy**	127 (18.4)			61 (28.5)			66 (13.8)			**<.001**
** Drainage**	71 (10.3)			55 (25.7)			16 (3.4)			**<.001**
** Perianal**	388 (6.9)			361 (52.2)			27 (0.5)			**<.001**
** Drainage**	147 (37.9)			136 (37.7)			11 (40.7)			.751
**Any hospitalization since diagnosis, *n* (%)**	2095 (39.1)	*n* = 5356		316 (48.8)	*n* = 647		1779 (37.8)	*n* = 4709		**<.001**
**Number of hospitalizations**		*n* = 2035			*n* = 313			*n* = 1722		.203
** Mean ± standard deviation**	2.11 ± 2.57			2.62 ± 3.62			2.02 ± 2.32			
** Median [min-max]**	1.00 [1-30]			1.00 [1-25]			1.00 [1-30]			
** 1-3**	1775 (87.2%)			262 (83.7%)			1513 (87.9%)			**<.001**
** 4-6**	183 (9.0%)			24 (7.7%)			159 (9.2%)			
** 7-9**	23 (1.1%)			5 (1.6%)			18 (1.0%)			
** 10+**	54 (2.7%)			22 (7.0%)			32 (1.9%)			
**Hospitalization rate (per patient-year)**	0.075			0.110			0.070			—
**Number of total relapses since diagnosis**	*n* = 4748			*n* = 566			*n* = 4182			**<.001**
** Mean ± standard deviation**	1.24 ± 2.64			1.64 ± 2.73			1.19 ± 2.63			
** Median (Q1-Q3)**	0.00 (0.00-2.00)			1.00 (0.00-2.00)			0.00 (0.00-2.00)			

Percentages are based on patients with information (*n*). Overall patients include UC, CD, and unclassified IBD patients. Percentages are based on patients with information (n column). *P*-values presented in bold indicate statistical significance.

Abbreviations: DTT, difficult-to-treat; IBD, inflammatory bowel disease; Max, maximum; Min, minimum; Q1, first quartile; Q3, third quartile.

a *P-*value from the Mann-Whitney test, for quantitative variables, and chi-square test for qualitative variables.

b Patients could report more than one type of surgery.

In addition, hospitalizations were more frequent among DTT patients, who also presented more relapses since diagnosis compared with the non‑DTT group.

### IBD treatment of DTT patients

**Figure 2:**
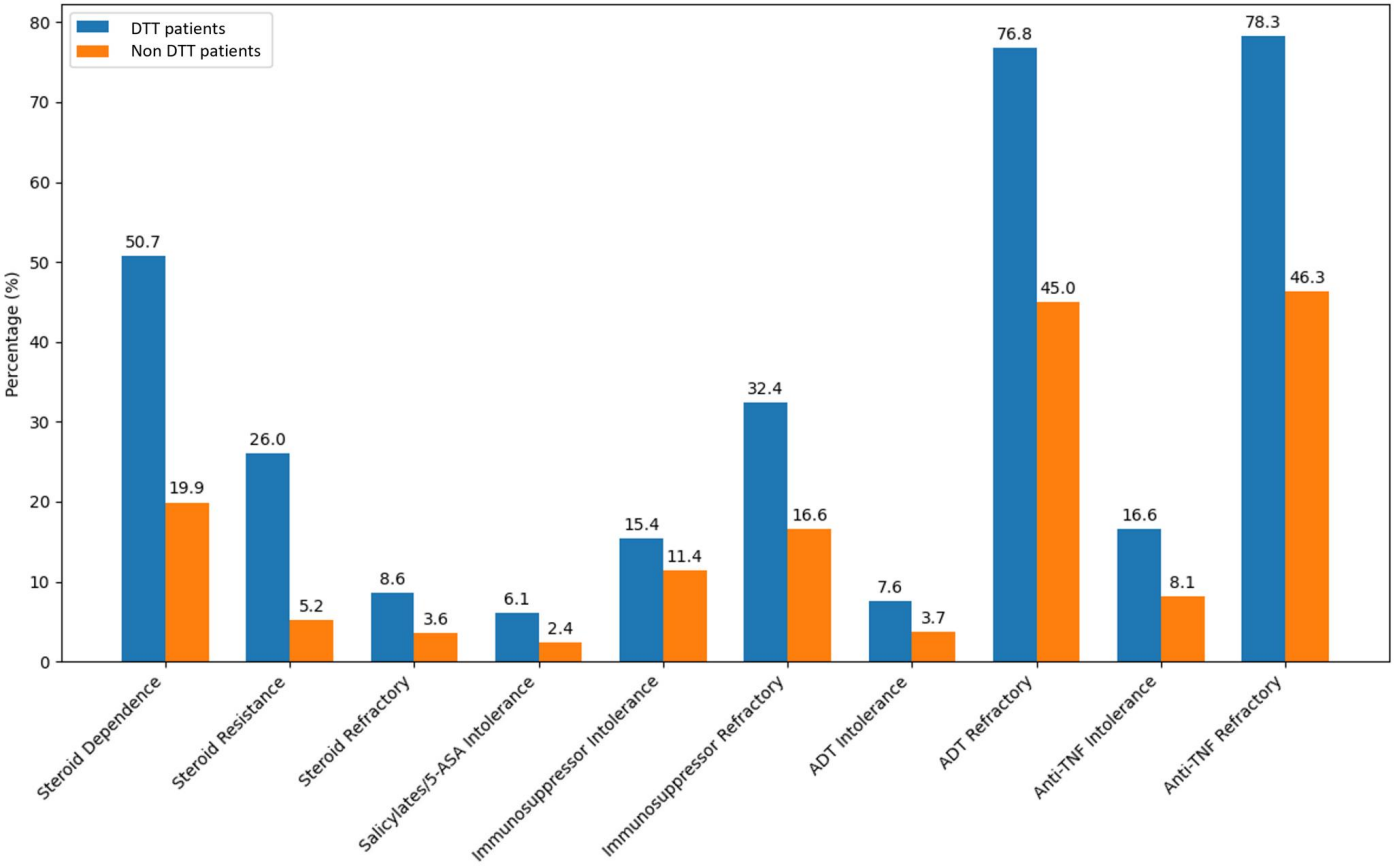
IBD treatment intolerance, resistance/refractory, and steroid dependence: DTT and non-DTT patients.

Among patients who received at least 1 ADT, 25.6% (*n* = 621) were identified as DTT, of whom 66.8% were treated exclusively with anti‑TNF agents (Table 5). Overall, DTT patients received more intensive therapy than non-DTT patients, as statistically significantly more DTT patients received ADTs (83.1%) and immunomodulators (64.1%). Use of anti‑TNF, anti‑interleukin, anti‑integrin therapies, and JAK inhibitors was significantly more common in the DTT group. Among DTT patients, the median (Q1–Q3) time until the first ADT was 2.5 (1–8) years. Treatment refractoriness occurred in 76.8% of DTT patients prescribed ADT (Figure 2). In addition, when comparing DTT patients referred from public vs. private institutions, the latter group had statistically more patients with 3 or more ADTs (26.6%) and meeting the DTT criteria “failure of 2+ ADT mechanisms” (23.4%) (Table S4).

**Table 5:** IBD treatment since diagnosis: DTT and non-DTT patients.

	Overall (*N* = 5699)		DTT group (*N* = 747)		Non-DTT group (*N* = 4952)		*P*-value^a^
**Pharmacological class and drug^b^, *n* (%)**	*n* = 5699		*n* = 747		*n* = 4952		
**Advanced drug therapy (ADT), *n* (%)**	2426 (42.6)		621 (83.1)		1805 (36.4)		**<.001**
** Adalimumab**	1089 (19.1)		320 (42.8)		769 (15.5)		—
** Certolizumab**	56 (1.0)		27 (3.6)		29 (0.6)		—
** Golimumab**	57 (1.0)		3 (0.4)		54 (1.1)		—
** Infliximab**	1316 (23.1)		415 (55.6)		901 (18.2)		—
** Tofacitinib**	35 (0.6)		22 (2.9)		13 (0.3)		—
** Ustekinumab**	268 (4.7)		134 (17.9)		134 (2.7)		—
** Vedolizumab**	360 (6.3)		98 (13.1)		262 (5.3)		—
** Other**	63 (1.1)		30 (4.0)		33 (0.7)		—
**Immunomodulators, *n* (%)**	2445 (42.9)		479 (64.1)		1966 (39.7		**<.001**
**Salicylates, *n* (%)**	3875 (68.0)		251 (33.6)		3624 (73.2)		**<.001**
**Steroids, *n* (%)**	1610 (28.3)		204 (27.3)		1406 (28.4)		.540
**Median (Q1-Q3) number of IBD treatments^c^**	2.0 (1.0-3.0)	*n* = 5635	3.0 (2.0-4.0)	*n* = 685	2.0 (1.0-3.0)	*n* = 4950	**<.001**
**Median (Q1-Q3) number of ADT^d^**	0.0 (0.0-1.0)	*n* = 5635	1.0 (1.0-2.0)	*n* = 683	0.0 (0.0-1.0)	*n* = 4952	**<.001**
** 0**	3210 (57.0%)		63 (9.2%)		3147 (63.6%)		
** 1**	1825 (32.4%)		355 (52.0%)		1470 (29.7%)		
** 2**	414 (7.3%)		132 (19.3%)		282 (5.7%)		
** 3**	157 (2.8%)		108 (15.8%)		49 (1.0%)		
** >3**	29 (0.5%)		25 (3.7%)		4 (0.1%)		
**ADT mechanism of action^b,d^, *n* (%)**	*n* = 5699		*n* = 747		*n* = 4952		
** Anti-TNF**	2139 (37.5)		594 (79.5)		1545 (31.2)		**<.001**
** 1**	1759 (82.2)		429 (72.2)		1330 (86.1)		—
** 2**	354 (16.5)		158 (26.6)		196 (12.7)		—
** ≥3**	26 (1.2)		7 (1.2)		19 (1.2)		—
** Anti-integrin**	373 (6.5)		103 (13.8)		270 (5.5)		**<.001**
** Anti-interleukin**	288 (5.1)		144 (19.3)		144 (2.9)		**<.001**
** Janus kinase (JAK) inhibitors**	56 (1.0)		31 (4.1)		25 (0.5)		**<.001**
**ADT mechanism of action options^d^, *n* (%)**	*n* = 2422		*n* = 621		*n* = 1801		
** Only anti-TNF**	1785 (73.7)		415 (66.8)		1370 (76.1)		**<.001**
** 1**	1540 (86.3)		336 (81.0)		1204 (87.9)		—
** 2**	224 (12.5)		73 (17.6)		151 (11.0)		—
** ≥3**	21 (1.2)		6 (1.4)		15 (1.1)		—
** Only anti-integrin**	182 (7.5)		6 (1.0)		176 (9.8)		—
** Only anti-IL**	76 (3.1)		14 (2.3)		62 (3.4)		—
** Only JAK inhibitors**	11 (0.5)		0		11 (0.6)		—
** Anti-TNF + anti-integrin**	131 (5.4)		43 (6.9)		88 (4.9)		—
** Anti-TNF + anti-IL**	152 (6.3)		76 (12.2)		76 (4.2)		—
** Anti-TNF + JAK inhibitors**	14 (0.6)		4 (0.6)		10 (0.6)		—
** Anti-integrin + anti-IL**	8 (0.3)		4 (0.6)		4 (0.2)		—
** Anti-integrin + JAK inhibitors**	1 (0.0)		0		1 (0.1)		—
** Anti-IL + JAK inhibitors**	2 (0.1)		0		2 (0.1)		—
** Anti-TNF + anti-integrin + anti-IL**	32 (1.3)		32 (5.2)		0		—
** Anti-integrin + anti-IL + JAK inhibitors**	3 (0.1)		3 (0.5)		0		—
** Anti-TNF + anti-IL + JAK inhibitors**	9 (0.4)		9 (1.4)		0		—
** Anti-TNF + anti-integrin + JAK inhibitors**	10 (0.4)		9 (1.4)		1 (0.1)		—
** Anti-TNF + anti-integrin + anti-IL + JAK inhibitors**	6 (0.2)		6 (1.0)		0		—
**Years between diagnosis and start of first ADT**	*n* = 2122		*n* = 546		*n* = 1576		.195
** Mean ± standard deviation**	7.15 ± 10.65		6.76 ± 10.11		7.28 ± 10.83		
** Median (Q1-Q3)**	3.00 (1.00-9.00)		2.50 (1.00-8.00)		3.00 (1.00-9.00)		

Percentages are based on valid data from patients in the analysis dataset. *P*-values presented in bold indicate statistical significance.

Abbreviations: ADT, advanced drug therapy; CD, Crohn’s disease; DTT, difficult-to-treat; IBD, inflammatory bowel disease; IL, interleukin; JAK, Janus Kinase; *N*, number of patients; Q1, first quartile; Q3, third quartile; UC, ulcerative colitis.

a *P-*value from the Mann-Whitney test, for quantitative variables, and chi-square test for qualitative variables.

b Patients could report more than one treatment. Percentages calculated for all included patients (including those with missing information).

c Includes previous and current treatments.

d Only distinct ADTs (previous and current) were considered.

### Evaluation of other criteria related to DTT-IBD

Patients who required 3 or more ADTs (*n* = 205) were less frequently referred from public institutions (17.3%) and less often smoking at registry inclusion (7.2%), compared with those treated with fewer ADTs (Table S5). They also had a more severe and long-standing disease course, with longer disease duration, and both symptom onset and diagnosis occurred at a younger age compared with patients receiving fewer ADTs.

In addition, patients who received 3 or more ADTs presented a higher burden of comorbidities, particularly psychiatric (8.6%), skin (6.3%), hematological (3.4%), and urogenital (6.2%) conditions, along with a greater frequency of IBD‑related complications, IBD-related surgeries, and hospitalizations (Tables S6 and S7).

## Discussion

Information on IBD epidemiology in LATAM is still limited,^4^ despite the burden of moderate to severe IBD having been evaluated in some Latin American countries.^2^^,^^13^^,^^14^ This large, multicenter registry study provides one of the first estimates of DTT‑IBD prevalence in Latin America, applying the IOIBD consensus criteria to nearly 6000 patients with complete data. As this consensus definition is recent, estimates of the frequency of DTT-IBD in the overall IBD population are very scarce.^15^

We observed that 13% of all IBD patients were classified as DTT‑IBD, with a much higher proportion in CD than in UC. Among DTT‑CD patients, complex perianal disease was by far the most common criterion, unlike other European studies, where multidrug failure predominates. In two Italian tertiary centers, 24.8% of patients receiving ADT met at least one DTT-IBD criterion, of which 77% failed at least 2 mechanisms of action.^15^ The different distribution of DTT criteria between studies likely reflects a combination of true population differences, healthcare system factors, and methodological variations. In our study, 25.6% of patients with at least 1 ADT were classified as DTT, and 67% of DTT patients received only anti-TNFs. We also found that more DTT patients referred from private settings had undergone 3 or more ADTs, highlighting differences in ADT access compared with the public system—differences that likely did not occur in the tertiary centers included in Parigi et al.’s study, where the use of ADTs has been increasing. In addition, we propose that future research examine whether patients treated in public LATAM settings may require additional surgeries due to limited access to ADTs, which could lead them to meet the DTT criterion of postoperative recurrence more often.

The DTT-IBD criteria enable patient profiling of those who can benefit from additional medical care. DTT patients were mainly male, had a younger age at diagnosis and symptom onset, longer time until diagnosis, and more aggressive CD phenotypes (more penetrating cases and with ileocolonic or colonic locations), which were also identified by other studies.^15^^,^^16^

They also showed higher burdens of comorbidities, EIMs, and IBD complications, including fistulas, obstruction, perforation, and infections. Of note, approximately twice as many DTT patients had psychiatric comorbidities (about 8% of DTT patients), which can impact quality of life and increase the difficulty of managing these patients. IOIBD consensus also proposed the DTT criterion “presence of psychiatric comorbidities that interfere with IBD management,” which we did not evaluate, as we anticipated that missing information could affect the interpretation of these results in our retrospective registry-based study.^8^

Notwithstanding, DTT was associated with more surgeries, more ADTs (in terms of number of ADTs and number of mechanisms of action), and a high rate of refractoriness to therapy. However, we observed that most DTT-IBD patients (66.8%) received only anti-TNF agents (of whom most received only 1 anti-TNF and more than a quarter received 2 anti-TNF drugs), which may be due to access conditions to IBD treatments. It has been recognized that LATAM countries have heterogeneous access to specialized healthcare for IBD diagnosis and management.^4^^,^^17–19^ For that reason, IOIBD experts (although not achieving consensus agreement) and experts from our study group proposed other criteria potentially related to DTT-IBD.^8^ In our study, patients receiving 3 or more ADTs mirrored the DTT profile, but were less often treated in public systems, indicating access constraints.

### Clinical implications

The DTT‑IBD profile in Latin America likely reflects both severe disease phenotypes and structural barriers to diverse therapies, such as limited options beyond anti-TNFs in public settings. These barriers likely underestimate multidrug failure and amplify surgical and hospitalization risk, while the notable psychiatric comorbidity underscores unmet needs for integrated care. The DTT criteria may be used to guide referral to expert centers, prioritize advanced therapies, and identify high‑need patients even when full IOIBD criteria cannot be met. In addition, LATAM patients with a high-risk profile (ie, younger onset, ileocolonic/penetrating CD, and early complications) warrant closer management.

### Limitations and strengths

We recognize some relevant limitations of the study, namely:

- The retrospective cross‑sectional design hinders the ability to assess causality, treatment sequencing, or time‑dependent outcomes (eg, refractoriness), due to the missing data on treatment start/end dates and covariate timing.
- A high proportion of ineligible or unclassifiable patients due to missing information, as only 5699 of 9280 patients (≈61%) could be classified for DTT‑IBD. This introduces potential selection bias, as the analyzed population may not represent the full IBD population in the registries.
- We cannot rule out misclassification related to the DTT proxies used, particularly the assumption that all perianal cases were clinically complex and that patients receiving 3 or more advanced therapies had true treatment failure. Some therapy switches may occur for other reasons, such as adverse events or preference, which could lead to an overestimation of DTT‑IBD.
- On the other hand, the limited availability or reimbursement of advanced therapies (eg, in public systems) may lead to underestimation of DTT‑IBD, since patients who clinically need additional mechanisms of action may not meet the formal criteria simply due to lack of access and not due to disease characteristics.^4^^,^^18^
- Because some Latin American countries (eg, Chile) were not represented, the registries covered different time periods, and a large proportion of patients came from private settings, the generalizability of these findings across the region may be limited.

Despite these limitations, our study provides a characterization of DTT-IBD, the main risk factors, and overall information about hospitalizations, surgeries, and treatment experience in LATAM. This is the first multicenter and multinational study to use the consensus-defined IOIBD criteria for DTT-IBD applied to secondary data, providing insight into their validity and limitations for worldwide use.

## Conclusion

Our findings highlight that the unmet medical needs and burden of IBD management are heterogeneous in Latin America, especially when considering DTT-IBD patients and the limited access to advanced therapies. The initiation of more efficacious and safer therapies earlier in the disease course may help to modify it and reduce complications and healthcare costs. DTT-IBD criteria are a tool for patient management, and further research is needed to characterize risk factors for DTT-IBD and the impact of early intervention on these patients.

## Supplementary Material

otag058_Supplementary_Data

## Data Availability

The data that support the findings of this study are available from the corresponding author upon reasonable request.
