# Supplementary material for: Clinical characterization and healthcare burden of difficult-to-treat inflammatory bowel disease in Latin America: a real-world registry-based study
Source: Crohns Colitis 360. 2026 Jun 13;8(3):otag058. doi: 10.1093/crocol/otag058 (PMC13355588; doi:10.1093/crocol/otag058)
Supplement: otag058_Supplementary_Data [file otag058_supplementary_data.docx]

# Supplementary Data

Supplementary Table 1 – Characteristics of included patients and DTT classification, overall and by IBD type

|  | **Total  N=6417** | | **Ulcerative Colitis N=3572** | | **Crohn’s Disease  N=2778** | |
| --- | --- | --- | --- | --- | --- | --- |
|  |  | |  | |  | |
| **Female sex, n (%)** | 3603 (56.2%) | [n=6414] | 2136 (59.8%) | [n=3571] | 1440 (51.7%) | [n=2786] |
| **Disease duration (years), median (Q1-Q3)** | 9 (5-15) | [n=6206] | 9 (5-16) | [n=3482] | 9 (5-15) | [n=2668] |
| **Disease duration (years), mean ± sd** | 11.3 ± 8.6 | [n=6206] | 11.5 ± 8.4 | [n=3482] | 11.1 ± 8.8 | [n=2668] |
| **Country, n (%)** |  | |  | |  | |
| Argentina | 513 (8.0%) | | 378 (10.6%) | | 130 (4.7%) | |
| Brazil | 3656 (57.0%) | | 1692 (47.4%) | | 1934 (69.4%) | |
| Colombia | 103 (1.6%) | | 84 (2.4%) | | 18 (0.6%) | |
| Cuba | 73 (1.1%) | | 51 (1.4%) | | 22 (0.8%) | |
| Dominican Republic | 331 (5.2%) | | 130 (3.6%) | | 197 (7.1%) | |
| Ecuador | 122 (1.9%) | | 91 (2.5%) | | 22 (0.8%) | |
| Mexico | 1255 (19.6%) | | 953 (26.7%) | | 295 (10.6%) | |
| Peru | 31 (0.5%) | | 15 (0.4%) | | 16 (0.6%) | |
| Puerto Rico | 157 (2.4%) | | 43 (1.2%) | | 114 (4.1%) | |
| Uruguay | 19 (0.3%) | | 12 (0.3%) | | 7 (0.3%) | |
| Venezuela | 157 (2.4%) | | 123 (3.4%) | | 33 (1.2%) | |
|  |  | |  | |  | |
| **DTT-IBD classification, n (%)** | 747 (13.1%) | [n=5699] | 54 (1.6%) | [n=3347] | 693 (30.2%) | [n=2297] |
| *By individual DTT-IBD criteria, n (%)* |  | |  | |  | |
| Failure to 2+ ADT mechanisms | 110 (14.7%) | | 50 (92.6%) | | 60 (8.7%) | |
| Postoperative recurrence (CD patients) | 38 (5.1%) | | -- | | 38 (5.5%) | |
| Refractory pouchitis | 12 (1.6%) | | 4 (7.4%) | | 8 (1.2%) | |
| Complex perianal disease (CD patients) | 627 (83.9%) | | -- | | 627 (90.5%) | |
|  |  | |  | |  | |
| **DTT-IBD classification without perianal disease criteria, n (%)** | 158 (2.9%) | [n=5359] | 54 (1.6%) | [n=3343] | 104 (5.3%) | [n=1961] |
|  |  | |  | |  | |
| CD: Crohn’s disease; DTT: Difficult to treat; IBD: Inflammatory Bowel Disease; Q1: First quartile; Q3: Third quartile; SD: Standard deviation. | | | | | | |

Supplementary Table 2 – Logistic regression for baseline factors associated with DTT-CD (final model)

|  | **Odds Ratio** | **95%CI for OR** | **p-value** |  |
| --- | --- | --- | --- | --- |
|  |  |  |  |  |
| **Sex: Female vs Male** | 0.714 | [0.549; 0.929] | 0.012 |  |
|  |  |  |  |  |
| **Age at start of symptoms** | 0.967 | [0.957; 0.976] | <0.001 |  |
|  |  |  |  |  |
| **CD location at diagnosis** |  |  |  |  |
| Ileal vs Ileocolonic | 0.820 | [0.595; 1.132] | 0.228 |  |
| Colonic vs Ileocolonic | 1.962 | [1.416; 2.720] | <0.001 |  |
| Upper gastrointestinal tract isolated vs Ileocolonic | 1.165 | [0.496; 2.738] | 0.725 |  |
|  |  |  |  |  |
| CD: Crohn’s disease; OR: Odds ratio; 95%CI: 95% confidence interval; DTT: Difficult to treat.  The following variables were included in the initial model: Gender, age at IBD diagnosis, age at symptom onset, CD location at diagnosis, referral institution, time between onset of symptoms and diagnosis. | | | | |

Supplementary Table 3 – Logistic regression for baseline factors associated with DTT-UC (final model)

|  | **Odds Ratio** | **95%CI for OR** | **p-value** |  |
| --- | --- | --- | --- | --- |
|  |  |  |  |  |
| **Referral institution: Private vs Public** | 3.645 | [1.503; 8.838] | 0.004 |  |
|  |  |  |  |  |
| UC: Ulcerative Colitis; OR: Odds ratio; 95%CI: 95% confidence interval; DTT: Difficult to treat.  The following variables were included in the initial model: Gender, age at IBD diagnosis, age at symptom onset, UC extension at diagnosis, referral institution, time between onset of symptoms and diagnosis. | | | | |

Supplementary Table 4 – Characteristics of DTT patients by public vs. private referral institution

|  | **Total (n=416)** | | **Public (n=111)** | | **Private (n=305)** | | **p-value ^a)^** |
| --- | --- | --- | --- | --- | --- | --- | --- |
|  |  | |  | |  | |  |
| **Time between diagnosis and start of first ADT (years), median (Q1-Q3)** | 2 (1-8) | [n=336] | 2 (1-6) | [n=88] | 2 (1-9.5) | [n=248] | 0.127 |
|  |  |  |  |  |  |  |  |
| **Patients with at least 3 ADTs, n (%)** | 89 (21.4%) | | 8 (7.2%) | | 81 (26.6%) | | **<0.001** |
|  |  | |  | |  | |  |
| **DTT-IBD criteria, n (%)** |  |  |  |  |  |  |  |
| Failure of 2+ ADT mechanisms | 81 (19.6%) | [n=414] | 10 (9.1%) | [n=110] | 71 (23.4%) | [n=304] | **0.001** |
| Postoperative recurrence (CD patients) | 33 (18.4%) | [n=179] | 14 (24.6%) | [n=57] | 19 (15.6%) | [n=122] | 0.149 |
| Refractory pouchitis | 10 (2.5%) | [n=397] | 3 (2.8%) | [n=109] | 7 (2.4%) | [n=288] | 1.000 * |
| Complex perianal disease (CD patients) | 323 (86.4%) | [n=374] | 93 (88.6%) | [n=105] | 230 (85.5%) | [n=269] | 0.437 |
|  |  | |  | |  | |  |
| Note: Percentages are based on patients with information [n column].  ADT: Advanced Drug Therapy; IBD: Inflammatory Bowel Disease; UC: Ulcerative Colitis; CD: Crohn’s disease; DTT: Difficult to treat. n = Number of patients without missing information; Q1=First quartile; Q3= Third quartile.  a) p-values from the Mann-Whitney test, for quantitative variables, and chi-square test for qualitative variables, except * Fisher-exact-test. | | | | | | | |

Supplementary Table 5 – Demographic and clinical characteristics of patients with at least 3 ADTs vs. patients with less than 3 ADTs

|  | **Overall  (N=6108)** | **n** | **Patients with 3+ADTs (N=205)** | **n** | **Patients with less than 3 ADTs (N=5903)** | **n** | **p-value ^a)^** |
| --- | --- | --- | --- | --- | --- | --- | --- |
|  |  |  |  |  |  |  |  |
| **Age at IBD diagnosis (years), median (Q1-Q3)** | 33 (24-46) | 5896 | 27 (20-40) | 194 | 33 (24-47) | 5702 | **<0.001** |
| **Disease duration (years), median (Q1-Q3)** | 9 (5-15) | 5898 | 12 (9-17) | 195 | 9 (5-15) | 5703 | **<0.001** |
| **Age at symptom onset (years), median (Q1-Q3)** | 32 (23-45) | 5714 | 27 (19-39) | 180 | 32 (23-45) | 5534 | **<0.001** |
| **Female sex, n (%)** | 3431 (56.2%) | 6105 | 109 (53.2%) | 205 | 3322 (56.3%) | 5900 | 0.374 |
| **Family history (1^st^ grade) of IBD, n (%)** | 59 (1.9%) | 3026 | 2 (3.7%) | 54 | 57 (1.9%) | 2972 | 0.284 * |
| **Current smokers, n (%)** | 691 (12.0%) | 5781 | 14 (7.2%) | 195 | 677 (12.1%) | 5586 | **0.037** |
| **Private referral institution, n (%)** | 2569 (63.0%) | 4077 | 105 (82.7%) | 127 | 2464 (62.4%) | 3950 | **<0.001** |
| **CD location at diagnosis, n (%)** |  | 2452 |  | 138 |  | 2314 | **0.002** |
| Ileal ^b)^ | 803 (32.7%) |  | 28 (20.3%) |  | 775 (33.5%) |  |  |
| Colonic  ^b)^ | 530 (21.6%) |  | 29 (21.0%) |  | 501 (21.7%) |  |  |
| Ileocolonic  ^b)^ | 1056 (43.1%) |  | 79 (57.2%) |  | 977 (42.2%) |  |  |
| Upper gastrointestinal tract isolated | 63 (2.6%) |  | 2 (1.4%) |  | 61 (2.6%) |  |  |
| **CD phenotype at diagnosis, n (%)** |  | 2280 |  | 128 |  | 2152 | **0.005** |
| Nonstricturing, nonpenetrating (B1) | 1166 (51.1%) |  | 49 (38.3%) |  | 1117 (51.9%) |  |  |
| Stricturing (B2) | 650 (28.5%) |  | 41 (32.0%) |  | 609 (28.3%) |  |  |
| Penetrating (B3) | 464 (20.4%) |  | 38 (29.7%) |  | 426 (19.8%) |  |  |
| **Modifier p (perianal disease), n (%)** | 443 (17.1%) | 2591 | 41 (28.7%) | 143 | 402 (16.4%) | 2448 | **<0.001** |
| **UC extension at diagnosis, n (%)** |  | 3356 |  | 60 |  | 3296 | **<0.001** |
| Extensive colitis (E3) | 1648 (49.1%) |  | 47 (78.3%) |  | 1601 (48.6%) |  |  |
| Left-sided colitis (E2) | 929 (27.7%) |  | 12 (20.0%) |  | 917 (27.8%) |  |  |
| Proctitis (E1) | 779 (23.2%) |  | 1 (1.7%) |  | 778 (23.6%) |  |  |
|  |  |  |  |  |  |  |  |
| Note: Percentages are based on patients with information (n column). Overall patients include UC, CD, and unclassified IBD patients.  ADT: Advanced Drug Therapy; IBD: Inflammatory Bowel Disease; UC: Ulcerative Colitis; CD: Crohn’s disease; DTT: Difficult to treat. n: Number of patients without missing information; Q1: First quartile; Q3: Third quartile.  a) p-values from the Mann-Whitney test, for quantitative variables, and chi-square test for qualitative variables, except * Fisher-exact-test.  b) with or without involvement of upper gastrointestinal tract – CD location by overall patients: Ileal + upper gastrointestinal n=16, Colonic + Upper gastrointestinal tract n=4, Ileocolonic + Upper gastrointestinal tract n=16. | | | | | | | |

Supplementary Table 6 – Comorbidities, Complications, and EIMs: patients with at least 3 ADTs vs. patients with less than 3 ADTs

|  | **Total (n=6108)** | **With at least 3 ADTs (n=205)** | **With less than  3 ADTs (n=5903)** | **p-value ^a)^** |  |
| --- | --- | --- | --- | --- | --- |
|  |  |  |  |  |  |
| **Comorbidities, n (%)** |  |  |  |  |  |
| Musculoskeletal | 431 (7.2%) | 16 (7.8%) | 415 (7.2%) | 0.709 |  |
| Cardiovascular | 913 (16.4%) | 27 (14.2%) | 886 (16.5%) | 0.398 |  |
| Neurologic | 78 (1.5%) | 1 (0.6%) | 77 (1.5%) | 0.523 * |  |
| Psychiatric | 202 (4.0%) | 14 (8.6%) | 188 (3.9%) | **0.002** |  |
| Skin | 132 (2.5%) | 11 (6.3%) | 121 (2.4%) | **0.005 *** |  |
| Hepato/Gastrointestinal | 276 (5.5%) | 11 (6.8%) | 265 (5.4%) | 0.455 |  |
| Hematologic | 55 (1.1%) | 6 (3.4%) | 49 (1.0%) | **0.010*** |  |
| Urogenital | 119 (2.4%) | 10 (6.2%) | 109 (2.2%) | **0.005*** |  |
| Respiratory | 98 (1.9%) | 3 (1.9%) | 95 (1.9%) | 1.000 * |  |
| Endocrine | 747 (13.5%) | 31 (16.3%) | 716 (13.4%) | 0.240 |  |
| Oncology | 104 (1.9%) | 4 (2.3%) | 100 (1.9%) | 0.582 * |  |
| Other | 312 (5.8%) | 14 (8.0%) | 298 (5.7%) | 0.206 |  |
| **Any comorbidity, n (%)** | n=5458 | n=187 | n=5271 | **0.002** |  |
| No | 3166 (58.0%) | 88 (47.1%) | 3078 (58.4%) |  |  |
| Yes | 2292 (42.0%) | 99 (52.9%) | 2193 (41.6%) |  |  |
|  |  |  |  |  |  |
| **Complications of IBD since diagnosis ^b)^, n (%)** |  |  |  |  |  |
| Abdominal fistula/abscess | 97 (1.7%) | 9 (4.8%) | 88 (1.6%) | **0.004 *** |  |
| Perianal fistula/abscess | 378 (6.6%) | 40 (21.3%) | 338 (6.1%) | **<0.001** |  |
| Toxic megacolon | 21 (0.4%) | 1 (0.5%) | 20 (0.4%) | 0.504 * |  |
| Intestinal obstruction/perforation | 181 (3.2%) | 16 (8.5%) | 165 (3.0%) | **<0.001** |  |
| Colorectal cancer | 25 (0.4%) | 0 | 25 (0.5%) | 1.000 * |  |
| Infection | 168 (2.9%) | 23 (12.2%) | 145 (2.6%) | **<0.001** |  |
| Thromboembolic events | 95 (1.6%) | 5 (2.6%) | 90 (1.6%) | 0.236 * |  |
| Anemia | 689 (12.0%) | 16 (8.4%) | 673 (12.1%) | 0.116 |  |
| Fatigue | 587 (9.6%) | 31 (15.1%) | 556 (9.4%) | **0.006** |  |
| Other | 131 (2.2%) | 19 (10.0%) | 112 (2.0%) | **<.0001 *** |  |
| **Any IBD complication since diagnosis, n (%)** | n=5774 | n=188 | n=5586 | **<0.001** |  |
| No | 3909 (67.7%) | 73 (38.8%) | 3836 (68.7%) |  |  |
| Yes | 1865 (32.3%) | 115 (61.2%) | 1750 (31.3%) |  |  |
|  |  |  |  |  |  |
| **Extraintestinal manifestations ^c)^, n (%)** |  |  |  |  |  |
| Articular | 1469 (24.1%) | 72 (35.1%) | 1397 (23.7%) | **<0.001** |  |
| Arthralgia/arthritis | 1230 (20.1%) | 61 (29.8%) | 1169 (19.8%) | **<0.001** |  |
| Axial articular | 421 (6.9%) | 18 (8.8%) | 403 (6.8%) | 0.278 |  |
| Sacroiliitis | 119 (2.0%) | 6 (2.9%) | 113 (1.9%) | 0.300 * |  |
| Ankylosing spondylitis | 324 (5.4%) | 13 (6.4%) | 311 (5.4%) | 0.530 |  |
| Primary sclerosing cholangitis | 122 (2.0%) | 7 (3.4%) | 115 (1.9%) | 0.130 * |  |
| Skin | 164 (2.7%) | 4 (2.0%) | 160 (2.7%) | 0.509 |  |
| Pyoderma gangrenosum | 62 (1.0%) | 2 (1.0%) | 60 (1.0%) | 1.000 * |  |
| Erythema nodosum | 101 (1.7%) | 3 (1.5%) | 98 (1.7%) | 1.000 * |  |
| Uveitis | 134 (2.2%) | 8 (3.9%) | 126 (2.1%) | 0.090 * |  |
| Oral ulcers | 124 (2.1%) | 9 (4.4%) | 115 (2.0%) | **0.038 *** |  |
| **Any extraintestinal manifestation, n (%)** | n=6108 | n=205 | n=5903 | **<0.001** |  |
| No | 4370 (71.5%) | 120 (58.5%) | 4250 (72.0%) |  |  |
| Yes | 1738 (28.5%) | 85 (41.5%) | 1653 (28.0%) |  |  |
|  |  |  |  |  |  |
| Note: Percentages are based on valid information among patients on analysis dataset.  Note1: The information available in the database for this patient does not allow to determine the exact number of ADTs they received; however, it is known that they received at least three. For this reason, the patient was included in this analysis but excluded from the descriptive analysis.  ADT: Advanced Drug Therapy; EIM: Extraintestinal manifestations; IBD: Inflammatory Bowel Disease.  a) p-values from the chi-square test, except * Fisher-exact-test.  b) Patients could report more than one complication.  c) Patients could report more than one extraintestinal manifestation. The 'Skin' category includes patients with 'Pyoderma gangrenosum', 'Erythema nodosum' (CEMIC, GEDIIB, HPUC, PANCCO) or 'Tipo MEI - Piel' (Colombia). In the 'Arthralgia/arthritis' category, patients with 'Tipo MEI - Articular periférica' (Colombia) are included, in the 'Axial articular' category, patients with 'Sacroiliitis', 'Ankylosing spondylitis' (CEMIC, GEDIIB, HPUC, PANCCO) or 'Type MEI - Axial articular' (Colombia) are included, in the 'Articular' category, patients with 'Arthralgia/arthritis' or 'Axial articular' are included. | | | | | |

Supplementary Table 7 – IBD surgeries and hospitalizations: patients with at least 3 ADTs vs less than 3 ADTs

|  | **Total (n=6108)** | **With at least 3 ADTs (n=205)** | **With less than 3 ADTs (n=5903)** | **p-value ^a)^** |  |
| --- | --- | --- | --- | --- | --- |
|  |  |  |  |  |  |
| **Any surgery since diagnosis, n (%)** | n=6021 | n=204 | n=5817 | **<0.001** |  |
| No | 4685 (77.8%) | 90 (44.1%) | 4595 (79.0%) |  |  |
| Yes | 1336 (22.2%) | 114 (55.9%) | 1222 (21.0%) |  |  |
| Number of surgeries since diagnosis | n=1289 | n=111 | n=1178 | **<0.001** |  |
| Mean ± Standard deviation | 1.47 ± 0.95 | 1.95 ± 1.34 | 1.43 ± 0.89 |  |  |
| Median [min-max] | 1.00 [1-9] | 1.00 [1-7] | 1.00 [1-9] |  |  |
|  |  |  |  |  |  |
| 1 | 923 (71.6%) | 59 (53.2%) | 864 (73.3%) | **<0.0001*** |  |
| 2 | 223 (17.3%) | 25 (22.5%) | 198 (16.8%) |  |  |
| 3 | 91 (7.1%) | 12 (10.8%) | 79 (6.7%) |  |  |
| 4 | 27 (2.1%) | 9 (8.1%) | 18 (1.5%) |  |  |
| 5 | 10 (0.8%) | 2 (1.8%) | 8 (0.7%) |  |  |
| >5 | 15 (1.2%) | 4 (3.6%) | 11 (0.9%) |  |  |
|  |  |  |  |  |  |
| **Type of surgery ^b)^, n (%)** |  |  |  |  |  |
| Abdominal | 912 (15.5%) | 92 (46.0%) | 820 (14.4%) | **<0.001** |  |
| Perianal | 389 (6.6%) | 34 (17.0%) | 355 (6.2%) | **<0.001** |  |
|  |  |  |  |  |  |
| **Abdominal surgeries, n (%)** |  |  |  |  |  |
| Colectomy | 587 (64.4%) | 60 (65.2%) | 527 (64.3%) | 0.857 |  |
| Resection (small bowel and/or colon) | 802 (87.9%) | 86 (93.5%) | 716 (87.3%) | 0.085 |  |
| Ostomy | 173 (19.0%) | 19 (20.7%) | 154 (18.8%) | 0.664 |  |
| Drainage | 78 (8.6%) | 13 (14.1%) | 65 (7.9%) | **0.044** |  |
|  |  |  |  |  |  |
| **Perianal surgeries, n (%)** |  |  |  |  |  |
| Drainage | 148 (38.0%) | 16 (47.1%) | 132 (37.2%) | 0.257 |  |
|  |  |  |  |  |  |
| **Any hospitalization since diagnosis, n (%)** | n=5704 | n=190 | n=5514 | **<0.001** |  |
| No | 3373 (59.1%) | 71 (37.4%) | 3302 (59.9%) |  |  |
| Yes | 2331 (40.9%) | 119 (62.6%) | 2212 (40.1%) |  |  |
| Number of hospitalizations since diagnosis | n=2268 | n=118 | n=2150 | 0.820 |  |
| Mean ± Standard deviation | 2.23 ± 2.77 | 2.45 ± 3.06 | 2.21 ± 2.76 |  |  |
| Median [min-max] | 1.00 [1-30] | 1.00 [1-20] | 1.00 [1-30] |  |  |
|  |  |  |  |  |  |
| 1-3 | 1948 (85.9%) | 99 (83.9%) | 1849 (86.0%) | NA |  |
| 4-6 | 217 (9.6%) | 10 (8.5%) | 207 (9.6%) |  |  |
| 7-9 | 31 (1.4%) | 2 (1.7%) | 29 (1.3%) |  |  |
| 10+ | 72 (3.2%) | 7 (5.9%) | 65 (3.0%) |  |  |
|  |  |  |  |  |  |
| **Hospitalization rate (per patient-year)** | 0.082 | 0.113 | 0.081 | -- |  |
|  |  |  |  |  |  |
| **Number of total relapses since diagnosis** | n=5114 | n=158 | n=4956 | **<0.001** |  |
| Mean ± Standard deviation | 1.29 ± 2.75 | 3.37 ± 3.50 | 1.22 ± 2.70 |  |  |
| Median (Q1-Q3) | 0.00 (0.00-2.00) | 3.00 (1.00-4.00) | 0.00 (0.00-2.00) |  |  |
|  |  |  |  |  |  |
| \| Note: Percentages are based on valid information among patients on analysis dataset.  Note: The information available in the database for this patient does not allow to determine the exact number of ADTs they received; however, it is known that they received at least three. For this reason, the patient was included in this analysis but excluded from the descriptive analysis.  ADT: Advanced Drug Therapy; IBD: Inflammatory Bowel Disease; UC: Ulcerative Colitis; CD: Crohn’s disease. n: Number of patients; Q1: First quartile; Q3: Third quartile; Min: Minimum; Max: Maximum.  a) p-values from the Mann-Whitney test, for quantitative variables, and chi-square test for qualitative variables, except * Fisher-exact-test.  b) Patients could report more than one type of surgery. \| \| --- \| | | | | | |
